# Mixing Performance of a 3D Micro T-Mixer with Swirl-Inducing Inlets and Rectangular Constriction

**DOI:** 10.3390/mi9050199

**Published:** 2018-04-24

**Authors:** Jinxin Zhang, Xiaoping Luo

**Affiliations:** School of Mechanical and Automotive Engineering, South China University of Technology, Guangzhou 510641, China; meandyzhang@mail.scut.edu.cn

**Keywords:** microfluidics, 3D T-mixer, mixing index, vortices, chaotic advection

## Abstract

In this paper, three novel 3D micro T-mixers, namely, a micro T-mixer with swirl-inducing inlets (TMSI), a micro T-mixer with a rectangular constriction (TMRC), and a micro T-mixer with swirl-inducing inlets and a rectangular constriction (TMSC), were proposed on the basis of the original 3D micro T-mixer (OTM). The flow and mixing performance of these micromixers was numerically analyzed using COMSOL Multiphysics package at a range of Reynolds numbers from 10 to 70. Results show that the three proposed 3D micro T-mixers have achieved better mixing performance than OTM. Due to the coupling effect of two swirl-inducing inlets and a rectangular constriction, the maximum mixing index and pressure drop appeared in TMSC among the four micromixers especially; the mixing index of TMSC reaches 91.8% at *Re* = 70, indicating that TMSC can achieve effective mixing in a short channel length, but has a slightly higher pressure drop than TMSI and TMRC.

## 1. Introduction

In recent years, microfluidic systems have received great attention for their extensive applications involving sample preparation and analysis, flow chemistry, drug delivery, and chemical and biological analysis [1,2,3,4,5]. The micromixer, as an important component of these applications, realizes the mixing of the fluid samples [6,7]. The low Reynolds number makes the flow in the micromixers laminar, and therefore the mixing processes are dominated by molecular diffusion. Mixing processes mainly relying on molecular diffusion require considerable mixing time and a long channel length, which results in a large pressure drop and high costs. Therefore, how to achieve efficient and rapid mixing has been a matter of urgent and in-depth study, especially for low Reynolds numbers.

Micromixers can be divided into two categories: active and passive. Active micromixers are designed to control the flow and accelerate mixing by external energy sources, such as acoustic actuation [8,9,10], thermal actuation [11], electric fields [12,13], and magnetic fields [14,15,16]. Active micromixers show excellent mixing capabilities, but a drawback is their fabrication complexity. On the other hand, passive micromixers are designed to facilitate the mixing process by using special channel configurations to increase the interface of fluids. Compared with active micromixers, passive micromixers have many advantages such as less expense, no complex control units, no additional power input, the simplicity of their integration into microfluidic system, and suitability for disposable applications. However, the fluid interface modulation of a passive micromixer is much more difficult than an active micromixer, so improvement of the mixing capability is the most important issue with a passive micromixer [17].

One of the simplest passive micromixers is the T-mixer, in which the height of two inlet channels is equal to the height of the mixing channel. At a low Reynolds numbers, the mixing in the T-mixers is slow. When *Re* increases to an extent (*Re* > 300) [18,19,20], rapid mixing can be achieved for a T-mixer because the fluids coming from the inlets can generate an asymmetric flow at the entrance of the mixing channel. Therefore, in order to obtain efficient mixing at an extensive range of Reynolds numbers, many researchers have studied various approaches to micromixer design, including bent or curved channel arrangement [21,22,23,24], wall structures [25,26,27], obstacles within the channel [28,29], and modified T-shaped channels [30,31].

Cortes-Quiroz et al. [32] introduced a 3D T-mixer, in which the depth of two inlet channels was half of the depth of the mixing channel and the position of two inlet channels was different in terms of the direction of the mixing channel depth. The mixing performance of different configurations of the 3D T-mixer was numerically investigated. The design of micromixers could generate transversal vortices in the mixing channel that enhance the mixing performance. Cortes-Quiroz et al. [33] numerically and experimentally studied the mixing performance of the 3D T-mixer and a typical T-mixer. They found that the 3D T-mixer provided much higher levels of mixing compared to the typical T-mixer, while presenting lower pressure drop and shear stress in the outlet channel in a *Re* range of 10–250. Cortes-Quiroz et al. [34] conducted further research on the effect of the aspect ratio of the mixing channel on the flow characteristics and mixing performance in the 3D T-mixer. The results showed that the addition of the width of the mixing channel could significantly improve the mixing performance of the 3D T-mixer. Ansari et al. [35] experimentally investigated the mixing performance of the typical T-mixer and the novel 3D T-mixer, which they called a T-mixer with non-aligned inputs. The Reynolds number varied in the range of 10–70. The results indicated that the swirl flow was produced by the fluid coming from two inlet channels of the 3D T-junction, which could enhance mixing even at low Reynolds numbers. Rabani et al. [36] studied the effect of lamination of inlet flows in the 3D T-mixer on the mixing efficiency. The lamination in the inflow can effectively increase the interface of the fluids and strengthen the vortex at the entrance of the mixing channel. Unlike simple T-mixers, the 3D T-mixer is effective at improving the mixing performance even at low Reynolds numbers (*Re* ≥ 10). However, the high value of mixing index for the above micromixers are up to 0.7, though *Re* values are up to 300 or the inputs use lamination of two fluids. In order to complete more efficient mixing with a minimal residence time, applying design modification for 3D T-mixer will find greater practical applications for the main microfluidic systems.

Moreover, some researchers conducted extensive studies on the combination of various forms of micromixer structures to further enhance the mixing efficiency through stretching, folding, and break-up processes. Siconolfi et al. [37] studied the effect of geometry variations on the engulfment regime in micromixers by numerical simulation and stability analysis. The inclination of the inlet channels based on T-mixer is selected to identify the core of the instability. You et al. [38] numerically studied the mixing behavior of microfluidic mixers with variable inlet confluence angle. Increasing the confluence angle promotes the interaction of vortices in mixers to enhance mixing performance. Matsunaga and Nishino [39] presented a numerical study of the flow and mixing inside the T-mixer with two protrusions in the inlet channels. Compared to the typical T-mixer, this design can obtain a mixing improvement of more than twice due to that the obstacles in the inlet channels narrow and displace the fluid streams so they come into the T-junction at different vertical levels, intertwine and form a vortex flow in the mixing channel. Hossain et al. [40] numerically studied the mixing performance of a serpentine micromixer with non-aligned input channels. The serpentine micromixer with non-aligned input channels showed a higher mixing index than the serpentine micromixer with simple T-junction in the *Re* range from 0.1 to 90. Xia et al. [41] numerically and experimentally investigated the effects of gaps and baffles on the flow and mixing. Different locations of the gaps and baffles along the mixing channel were set to analyze the synergistic effects of multiple vortices, abrupt constriction/expansion, and twice split/recombine on the mixing performance. The results showed that the constriction and expansion at the beginning of the mixing channel was good for enhancing the mixing efficiency. Lu et al. [42] numerically investigated the flow and mixing in a straight tube with an expanding/contracting cross section in the mixing channel. They found that the intensification effects of the combination of sudden expansion and sudden constriction on the mixing efficiency of the device could be overlapped. In this paper, a 3D micro T-mixer with the swirl-inducing inlet and rectangular constriction is proposed for rapid and efficient mixing in a range of Reynolds numbers from 10 to 70. The 3D micro T-mixers with three different configurations were tested by numerical simulation to determine the effect of the swirl-inducing inlet and rectangular constriction on flow characteristics and mixing in the 3D T-mixer. The mixing performance of the proposed micromixers was compared to the original 3D micro T-mixer.

## 2. Model Descriptions

### 2.1. Geometric Configurations

**Figure 1:**
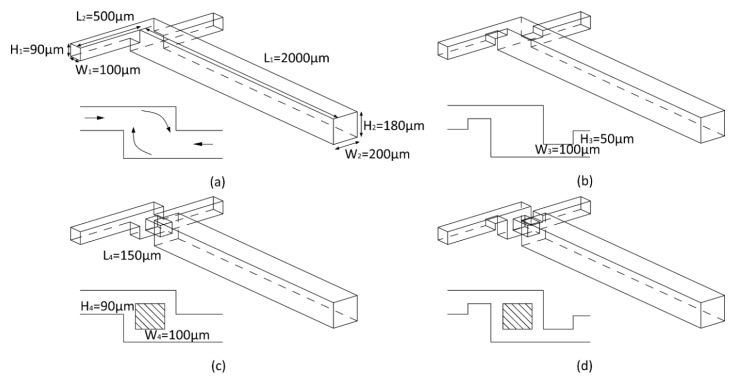
Isometric and top views of the 3D T-shaped micromixers: (**a**) OTM, (**b**) TMSI, (**c**) TMRC, (**d**) TMSC.

Figure 1a shows the schematic of the original 3D micro T-mixer (OTM), which is extracted from experimental setup of Ansari et al. [28]. In this mixer, the samples enter from inlet 1 and inlet 2 and are mixed at the entrance of the mixing channel due to the transversal vortices formed by the coming fluids from two inlet channels at different Z-coordinates. Figure 1b shows the 3D micro T-mixer with swirl-inducing inlets (TMSI), which is proposed in this work. A pair of obstacles placed in two inlet channels, which roll up the fluids and generate angular momentum, can slightly induce the swirling flow at the entrance of the mixing channel. Figure 1c shows the 3D micro T-mixer with rectangular constriction (TMRC). The rectangular constriction is placed at the entrance of the mixing channel. When flowing into constriction, the swirling flow becomes chaotic in the transversal section due to the smaller cross-sectional area, and after passing through the constriction, the swirling flow is decelerated and drawn away from the channel axis. Hence, the rectangular constriction can provide better mixing efficiency. A combination of the intensification of swirl-inducing inlet on the swirling flow and the constriction of rectangular constriction on the flow is likely to be effective in producing mixing performance, so the 3D micro T-mixer with swirl-inducing inlet and rectangular constriction (TMSC) is proposed as shown in Figure 1d. The reference values of these parameters for four micromixers are listed in Table 1.

### 2.2. Governing Equations and Boundary Conditions

In this study, the mixing performance of four micromixers is analyzed by using COMSOL Multiphysics 5.2 (COMSOL Inc., Stockholm, Sweden). This commercial code solves steady continuity, steady-incompressible Navier–Stokes and Convection–Diffusion equation by using the finite element to couple solver. In the analysis, four micromixers are simulated by numerically solving Equations (1)–(3), the specific equation is as follows:

(1) $$\mathrm{Steady}\;\mathrm{equation}:\nabla \cdot \vec{V}=0$$

(2) $$\mathrm{Navier}–\mathrm{Stokes}\;\mathrm{equation}:(\vec{V}\cdot \nabla )\vec{V}=-\frac{1}{\rho }\nabla p+\nu \nabla^{2}\vec{V}\;$$

(3) $$\mathrm{Convection}–\mathrm{Diffusion}\;\mathrm{equation}:\left(V\cdot \nabla \right)C=\alpha \nabla^{2}C$$

where *V*, *ρ*, *p*, *C*, *α* and *ν* represent fluid velocity, fluid density, fluid pressure, the concentration of dye solution in same water, diffusivity coefficient and Kinematic viscosity, respectively.

In order to study mixing, the working fluid uses the diffusion constant of the dye solution. Inlet 1 and inlet 2 have molar concentrations of 0 mol∙m^−3^ and 1 mol∙m^−3^, respectively. The boundary conditions for the four micromixers are set as velocity inlet and pressure outlet. The walls are set as no slip boundary condition. The fixed velocity is set to two inlets. For comparison purposes, water is used as the carrier fluid. The dynamic viscosity, density, and diffusion coefficient of the working fluid at 25 °C are 10^3^ kg s/m, 10^3^ kg/m^3^, and 1.5 × 10^9^ m^2^/s [42], respectively.

To investigate quantitatively the mixing performance of the micromixer, the mixing index of the species at a cross section is calculated as follows [43]:

(4) $$M=1-\sqrt{\frac{1}{N}\overset{N}{\underset{i=1}{\sum }}{\left(\frac{c_{i}-\bar{c}}{\bar{c}}\right)}^{2}}$$

where *M* is the mixing index, *N* is the total number of sampling points, *c_i_* and *c* are normalized concentration and expected concentration, respectively. The mixing index varies from 0 to 1. The higher mixing index represents a better concentration and higher mixing performance.

The Reynolds number (*Re*) and hydraulic diameter (*D*_h_) are defined as follows:

(5) $$Re=\frac{\rho_{\mathrm{in}}u_{\mathrm{in}}D_{h}}{\mu_{\mathrm{in}}}$$

(6) $$D_{h}=\frac{2WH}{W+H}$$

where *ρ*_in_, *μ*_in_, and *u*_in_ are the density, dynamic viscosity, and velocity of the fluid at the microchannel inlet, respectively. *W* and *H* are the width and height of the microchannel, respectively.

## 3. Results and Discussion

### 3.1. Mesh Independency

**Figure 2:**
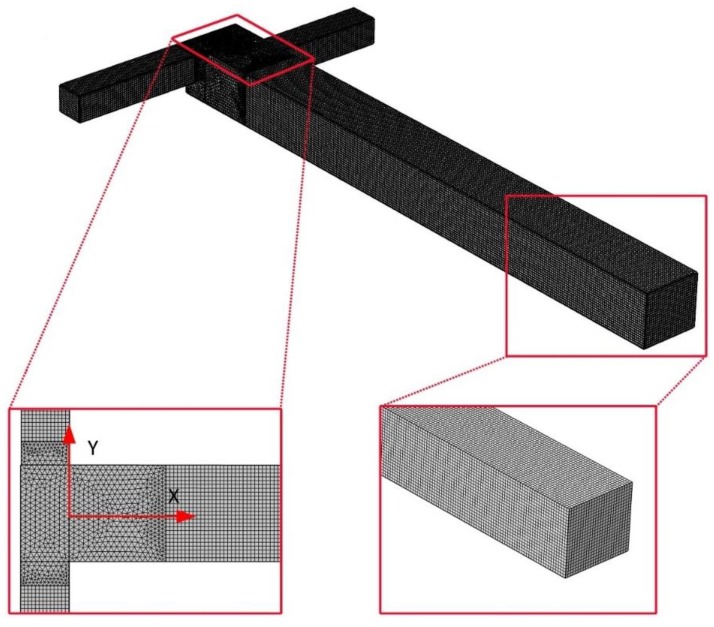
The mesh system of OTM with 277,511 elements.

**Figure 3:**
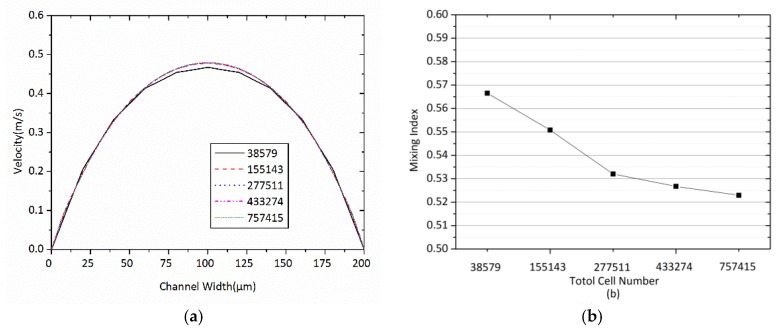
Mesh dependency test for OTM at *Re* = 50: (**a**) Local velocity profiles along the middle line at the outlet cross section with different mesh refinements, (**b**) Mixing index at the outlet cross section with different mesh refinements.

A mesh independency test is performed on the distribution of the velocity magnitude to determine the optimum number of grids for the micromixer. Five different mesh refinements, whereby the number of grids ranges from 38,579 to 757,415, are tested for OTM. Figure 2 shows a combination of structured and unstructured mesh. Figure 2 also shows the system of coordinates (X-Y), whose origin is located at the center of the mixing channel: *X* is the streamwise if the mixing channel; *Y* is the direction of normal to *X* in the plane of the inlet channels. The test is performed at *Re* = 50. Figure 3 shows the local velocity profiles along the middle line at the outlet, which are obtained from simulations with the five different mesh refinements. Beyond the node number of 155,143, the influence of increasing the node number on the accuracy of the results is negligible. In addition, in order to assess the grid sensitivity, the five case studies with total cell numbers of 38,579, 155,143, 277,511, 433,274 and 757,415 were carried out. The results of this inquiry are depicted in Figure 3b, where the mixing index is plotted against the total cell number. As is observed in Figure 2, after increasing the number of computational cells to 277,511 cells, the mixing index no longer changes. Thus, 277,511 is the minimum number of elements for meshing the presented OTM to obtain a mesh-independent solution; therefore, this mesh refinement is used for further simulations in this study. So, the minimum number of elements for meshing the presented TMSI, TMCB, and TMSC is 269,800, 234,902, and 227,126, respectively.

### 3.2. Validation

**Figure 4:**
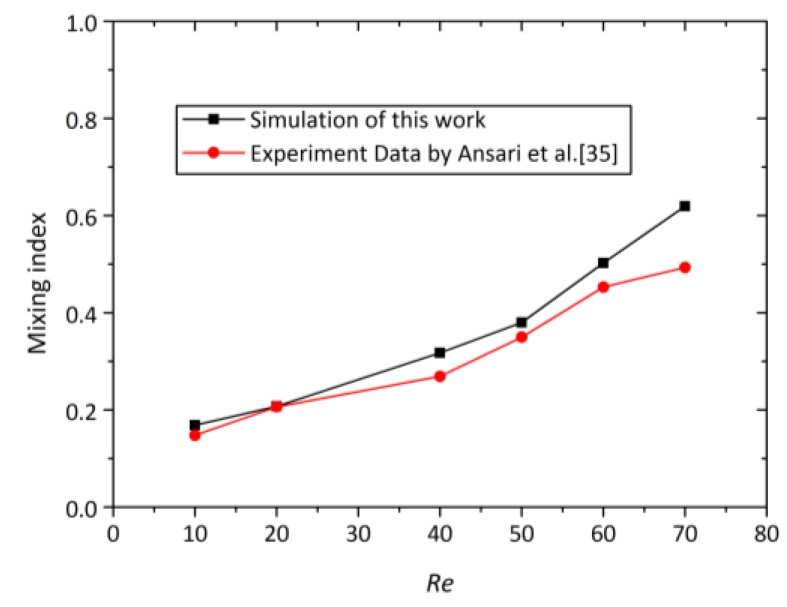
Comparison of the mixing index of the simulation results with the experimental data by Ansari et al. [35].

The simulation results were validated against Ansari et al.’s experimental data [35]. For this purpose, an identical simulation of Ansari’s study was run. The model geometry was extracted from Ansari’s experimental setup. The geometry of the original micromixer is similar to OTM as shown in Figure 1a except that the mixing channel (*L*_1_) of the original micromixer investigated experimentally by Ansari et al. is 7 mm, but the mixing channel of OTM is 2 mm. The experiment mixing index was captured and analyzed at the outlet of the micromixer. The mesh selected for this study was built up with the same structured and unstructured mesh in the same region comparing to the mesh refinement used in this study. Figure 4 presents a comparison of the mixing index on the outlet of the mixing channel in the range of the Reynolds number from 10 to 70. The mixing index is calculated by Equation (4). The tendency of numerical mixing index is in a good agreement with experiments of Ansari et al. [28]. There is deviation between numerical results and experimental data, which might come from fabrication errors and the difference in evaluating the mixing index. From the above results, the presented numerical method can predict the mixing performance of the proposed 3D T-mixers.

### 3.3. Mixing Index

**Figure 5:**
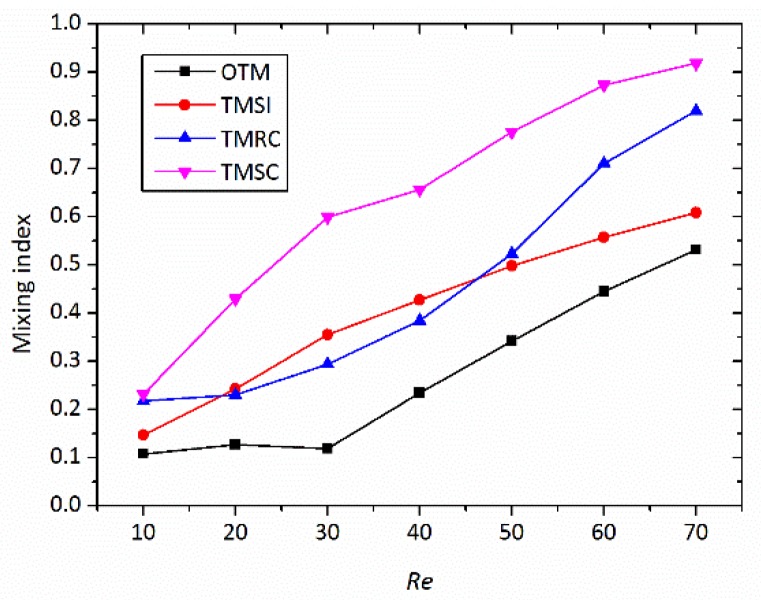
Variations of mixing index at the middle section of different micromixer at different Reynolds number.

Figure 5 depicts the variations of the mixing index versus Reynolds number for the four different micromixers. The mixing index is calculated at the middle section (*x* = 1.1 mm), which can represent the entire micromixer performance. As the Reynolds number grows, the mixing indices of all the micromixers increase correspondingly. At all the Reynolds numbers tested, the three proposed micromixers show much higher mixing efficiency than OTM. The swirl-inducing inlet structure in TMSI further enhances the swirling flow induced by two inlets at different horizontal planes, which is why TMSI has a higher mixing index than OTM. The constriction causes the constriction and expansion of the fluids in the mixing channel, which cause transversal mass transport to enhance the mixing performance of the micromixer. The mixing index of TMSC is always the highest among three novel micromixers, especially at *Re* = 70, when the mixing index of TMSC reaches about 91.8%, but TMRC is about 82% and TMSI is only 60.8%. This indicates that the enhancement of the asymmetrical obstacles and rectangular constriction on the mixing index of the 3D micromixer is superposed. At *Re* = 10, the mixing indices of the four micromixers are not changing much, i.e., the mixing indices of TMSC and TMRC are 10% higher than that of OTM. This illustrates that the transverse flow is not enough to induce advection, and therefore, fluid mixing only depends on molecular diffusion. Note that with the increase in the Reynolds number, the mixing indices of the three micromixers grow at a much faster rate than OTM. This is because, as the overall velocity becomes higher, the effect of the two studied structures on the advection is to enhance the mixing.

**Figure 6:**
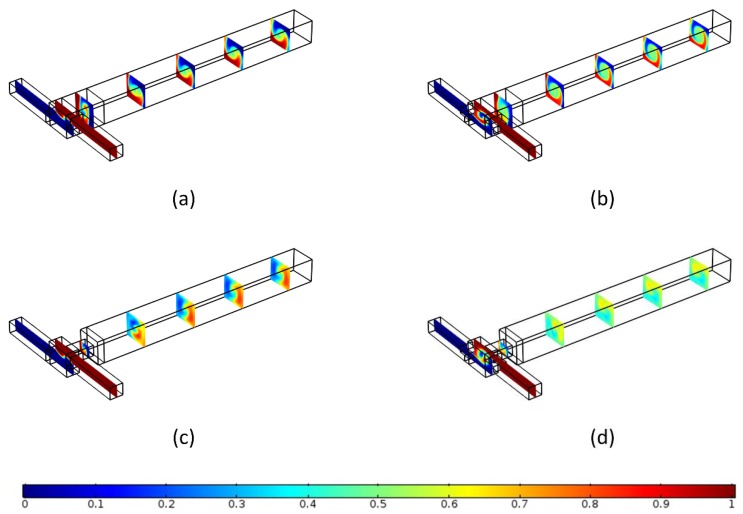
Streamlines and concentration distribution at each section in different micromixer at *Re* = 50 (**a**) OTM, (**b**) TMSI, (**c**) TMRC, (**d**) TMSC.

Due to various mixing mechanisms in the mixing channel such as splitting–recombining, twisting, advection, and vortices [44], the visualization of the flow and mixing is studied to analyze the mixing mechanisms of the four micromixers. Figure 6 presents the flow structure and mixing behavior in the four micromixers at *Re* = 50. The results reveal that, for all micromixers, the fluids coming from the two inlets generate a vortex at the entrance of the mixing channel, which is concentric to the mixing channel axis (*x*-axis). For OTM and TMSI, the swirl flow continues rotating in the mixing channel until it reaches the outlet section, as shown in Figure 6a,b. The rotation of the flow helps to reduce the diffusion path and increase the contact surface of fluids, which enhances mixing. TMSI have a higher mixing performance than OTM due to the asymmetric obstacles placed in the inlet creating a stronger vortex. On the other hand, after the flow runs through the rectangular constriction, the swirl flow becomes more chaotic, as shown in Figure 6c,d. This is because the frictional resistance and the pressure gradient led to the separation of the boundary layer, so the flow deflects away from the channel axis, which causes the flow to produce an obvious vortex. 

**Figure 7:**
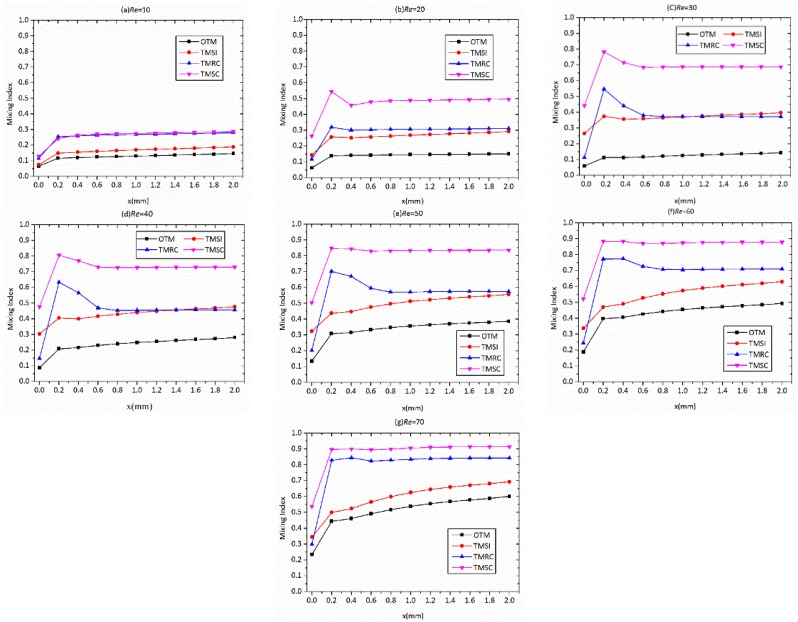
The comparison of the mixing index along the mixing channel for different micromixers at different Reynolds numbers: (**a**) *Re* = 10, (**b**) *Re* = 20, (**c**) *Re* = 30, (**d**), *Re* = 40, (**e**) *Re* = 50, (**f**) *Re* = 60, (**g**) *Re* = 70.

Figure 7 shows the development of the mixing index along the channel length for OTM, TMSI, TMRC, and TMSC at different Reynolds numbers, viz., 10, 20, 30, 40, 50, 60, and 70, respectively. The cross section is plotted along the channel length (*x* = 0, 0.2, 0.4, 0.6, 0.8, 1.0, 1.2, 1.4, 1.6, 1.8, or 2.0 mm). At all Reynolds numbers, the mixing index of OTM along the channel length is lower than that of TMSI, TMRC, and TMSC. For all Reynolds numbers studied, owing to the high flow rate, the residence time is so much shorter that molecular diffusion at the interfaces would not help much with mixing. The effects of swirl-inducing inlets and rectangular contraction are utilized to increase the contact area and disturb the flow [40]. However, for *Re* = 10, the flow disturbance caused by the structure of all four micromixers is not strong to bring about more convection, so the mixing index of all four micromixers is low because of poor diffusion. The mixing curves of OTM and TMSI have almost the same changes, and the mixing index of TMSI is higher than that of OTM at each section. This is due to the swirl-inducing inlet producing greater vortices compared to OTM.

**Figure 8:**
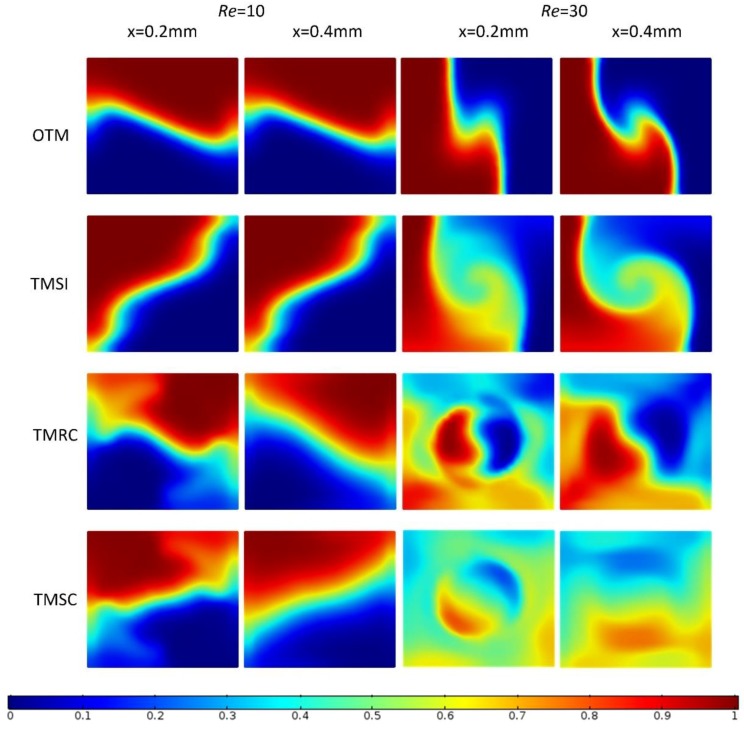
Concentration at each section at *x* = 0.2 mm and *x* = 0.4 mm for *Re* = 10 and 30.

At all Reynolds numbers, the mixing indices of the four micromixers grow rapidly from *x* = 0 mm to *x* = 0.2 mm; with the increase in Re, the magnitude of the growth becomes greater. After *x* = 0.4 mm, the mixing indices of four micromixers grow smoothly and slowly along the channel length. At *Re* = 10, the trends of the mixing index along the channel length are the same for the four micromixers. Obviously, at *Re* ≥ 20, the mixing indices of TMRC and TMSC along the channel length attain their maximum at *x* = 0.2 mm, decay to a steady value after *x* = 0.3 mm, then remain stable, as shown in Figure 7a–g). At *x* = 0.4 mm, mixing presents a slight reduction in cases with non-stable flows, which can be explained by the presence of local intermittent transversal and back flows, which has been proven to be valid by Cortes-Quiroz et al. [34]. Figure 8 shows the concentrations of two channel positions (*x* = 0.2 mm, 0.4 mm) at *Re* = 10 and *Re* = 30 for all the micromixers. The results indicate that at *Re* = 10, these fluid streams coming from the four micromixers are not capable of generating the vertical swirl flow, which is demonstrated by the concentration distribution of the four micromixers at *x* = 0.2 mm and 0.4 mm with *Re* = 10. The flow from the T-junction is in steady state, and the decrease in their kinetic energy along the channel length is not obvious. It is for this reason that the mixing indices of four micromixers at *x* = 0.2 mm and *x* = 0.4 mm are similar. At *Re* = 30, the flow of the four micromixers is too fast to generate the swirl flow at the entrance of the mixing channel. The intensity of the swirl flow depends on different three-dimensional structures of four micromixers. It is easy to see from Figure 8 that in descending order of enhancement of the three-dimensional structures for the swirl intensity at *x* = 0.2 mm in the mixing channel, they are TMSC, TMRC, TMSI, and OTM.

For TMRC and TMSC, the flow from the rectangular constriction produces the vortex intensification region and their kinetic energy decreases along the channel length. The vortex eventually goes down after some distance due to the action of fluid molecular viscosity. Hence, the mixing indices of TMRC and TMSC at *x* = 0.4 mm is lower than at *x* = 0.2 mm. At *Re* = 10, the mixing curves of TMRC and TMSC have almost the same changes. As the Reynolds number increases, the mixing performance of TMSC is better than TMRC. For TMRC and TMSC, the difference between the mixing indices at *x* = 0.2 mm and *x* = 0.4 mm becomes small, as shown in Figure 7b–g, because the higher flow rate allows the flow from the rectangular constriction to attain greater axial velocity, then to reduce the impact of fluid molecular viscosity on the kinetic energy.

Moreover, for all Reynolds numbers, the mixing index of TMSC along the channel length is the highest among the four micromixers. This is due to the combination of swirl-inducing inlet and rectangular constriction, which provides more contact surface for the working fluid compared to the other micromixers. As expected from Figure 7e–g, with *Re* ≥ 50, TMSC obtains a maximum mixing index at *x* = 0.2 mm and remains constant after *x* = 0.4 mm. This indicates that TMSC can attain rapid and efficient mixing in the short mixing length. 

### 3.4. Flow Characteristics

**Figure 9:**
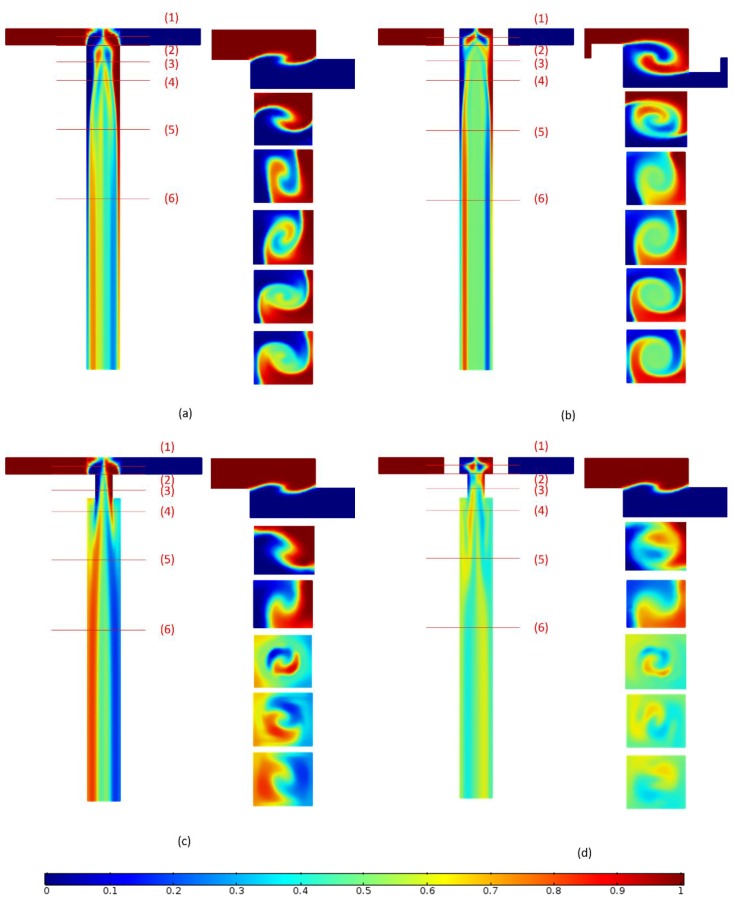
Concentration distribution at *Re* = 50 for (**a**) OTM, (**b**) TMSI, (**c**) TMRC, (**d**) TMSC. (1) *x* = −0.05 mm, (2) *x* = 0 mm, (3) *x* = 0.1 mm, (4) *x* = 0.2 mm, (5) *x* = 0.5 mm, (6) *x* = 1 mm.

The difference between the improvement of OTM and TMSI in mixing performance is due to the different swirl-inducing inlet. The difference between the improvements of TMRC and TMSC in mixing performance is due to the rectangular constriction. Therefore, Figure 9 shows a comparison of the concentration distribution of the four micromixers at *Re* = 50, and all planes are selected as the middle plane (*z* = 0.09 mm) along the height direction of the mixing channel and the cross sections at different distance (*x*-axis) of the mixing channel of four micromixers. Due to the two inlets being at different *Z*-coordinates, the fluid from the two inlets generates a vortex centered in the channel, which increases the contact surface of the working fluids. This phenomenon helps with enhancing the mixing performance. Hence, the cross section at *x* = −0.05 mm is set. The cross section at *x* = 0.1 mm is set because the rectangular constriction with the length of 0.15 mm placed at the entrance of the mixing channel in TMRC and TMSC is the reason for the improvement of TMRC and TMSC compared to OTM. In order to study a concentration distribution of different micromixers in the mixing channel, the other cross sections are also presented.

The swirl-inducing inlets of TMSI generate a greater vortex at *x* = −0.05 mm than OTM, and the velocity in the T-junction channel of TMSI more easily allows fluids to reach the opposite wall quickly as compared to OTM, as shown in Figure 9a,b. From Figure 9a,b, after the rotational flow comes into the mixing channel of OTM and TMSI, the flow continues rotating in the mixing channel until it reaches the outlet section, and the vortex of TMSI at each cross section is always greater than that of OTM. The rotation of the flow along the mixing channel increases the contact surface of fluids and narrows the striation thickness, which improves the mixing performance.

From Figure 9a,c, the concentration profile of TMRC at *x* = −0.05 mm is the same as that of OTM. This indicates that the rectangular constriction has less impact on the vortex caused by the T-junction channels. However, at *x* = 0.1 mm, the concentration distribution of TMRC in which the vortex becomes obvious is different from OTM, because the sudden constriction may restrain the development of local vortices to some extentand weaken the mixing strengthening effect. Compared to OTM, the fluid of TMRC at *x* = 0.5 mm becomes more active, due to the fluid flow away from the rectangular constriction. The suddenly expanded structure can make the vortex movement active with an increase in turbulence intensity and reduce the striation thickness along the mixing channel. It is worth mentioning that the concentration profile at each section of TMRC after *x* = 0.5 mm undergoes little change and is rotating along the mixing channel, as validated by Figure 6e.

The intersection area of the inlet channel and the mixing channel is as shown in Figure 9b. From Figure 9b,d, by comparing the concentration distribution of TMSI and TMSC at *x* = −0.05 mm, it is found that TMSC has more contacts for the two fluids at the entrance than TMSI. This may be due to the rectangular constriction, which impairs the flow from the T-junction to the mixing channel, promoting the development of the vortex caused by the T-junction structure and the swirl-inducing inlets.

At *x* = 0.1 mm, the concentration profile of TMSC is the same as that of TMRC because of the sudden constriction. However, for TMSC, two working fluids at the rectangular constriction obtain more sufficient mixing than TMRC, which is concluded from the concentration distribution of the middle plane shown in Figure 9c,d. The swirl-inducing inlets give the fluids a better mix, especially in the center of the mixing channel, and then the rotational fluids come into the rectangular constriction with the reduced striation thickness, which further enhances the mixing effect. Therefore, after *x* = 0.2 mm, the mixing performance of TMSC at each cross section is always higher than that of TMRC by comparing the concentration distribution at each plane as shown in Figure 9c,d. This indicates that the combination of the rectangular constriction and the swirl-inducing inlets can effectively enhance mixing and obtain high mixing performance even in the short distance.

**Figure 10:**
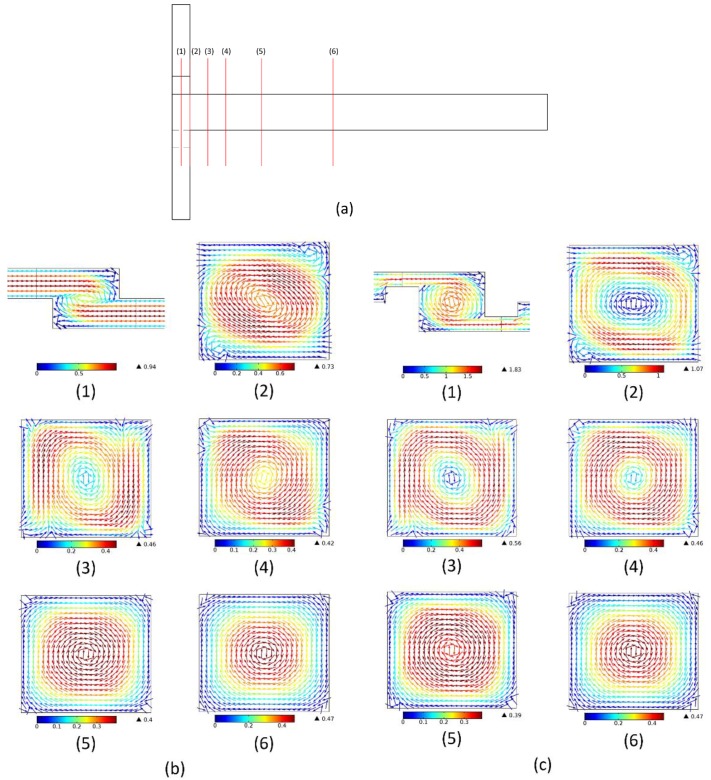

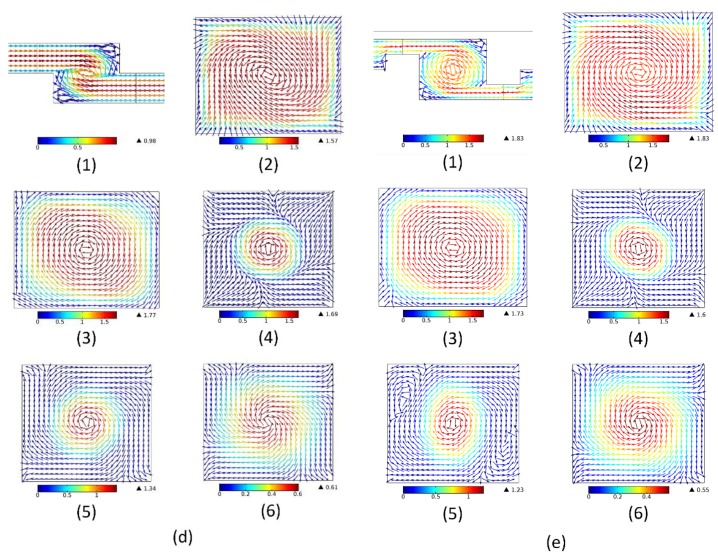
(**a**) The selected planes on four micromixers. (1) *x* = −0.05 mm, (2) *x* = 0 mm, (3) *x* = 0.1 mm, (4) *x* = 0.2 mm, (5) *x* = 0.5 mm, (6) *x* = 1 mm. Arrow surface of transversal flows at *Re* = 50 (**b**) OTM, (**c**) TMSI, (**d**) TMRC, (**e**) TMSC.

Figure 10 shows a comparison of transversal flows at selected plane of the four micromixers at *Re* = 50. A vortex is found in the figure of transversal flows at all *Re* numbers. In order to study the effect of the swirl-inducing inlets and the rectangular constriction on the mixing performance, the transversal flow at the cross sections along the mixing channel (at *x* = −0.05, 0, 0.1, 0.2, 0.4, 1 mm) should be considered. The planes are marked as shown in Figure 10a.

At *x* = −0.05 mm, the vortex on the cross section of TMSI is greater than that of OTM, and the magnitude of the velocity on the cross section of TMSI is bigger than OTM. After *x* = 0.1 mm, the distribution of the velocity on each plane of TMSI is the same as that of OTM, and the magnitude of the velocity on each plane of TMSI is bigger than that of OTM, but with the increase in the *x*-axis coordinates, the gap between the two becomes smaller. After *x* = 0.4 mm, the magnitude of the velocity of OTM and TMSI is close and undergoes little change. Considering that the curve of the mixing index along the channel length for TMSI is the same as that of OTM and high than that of OTM, as shown in Figure 7e, it can be seen that for TMSI and OTM, after some distance from the entrance of the mixing channel, the action of convective mixing becomes weak.

After *x* = 0 mm, the magnitude of the velocity on the section of TMRC is obviously higher than that of OTM, and after *x* = 0.2 mm, the distribution of the velocity on the section of TMRC, in which the fluid around the mixing channel rotates and moves to the center, is different to that of OTM. This indicates that after the fluids flow away from the rectangular constriction, the suddenly expanded structure can make the vortex movement active. Lastly, at all cross sections, the vortex in the center of the mixing channel of TMSC is bigger than that of TMRC, and the uniformity of the velocity of TMSC is better than that of TMRC. This is why TMSC has a higher mixing performance than TMRC, as shown in Figure 7e.

**Figure 11:**
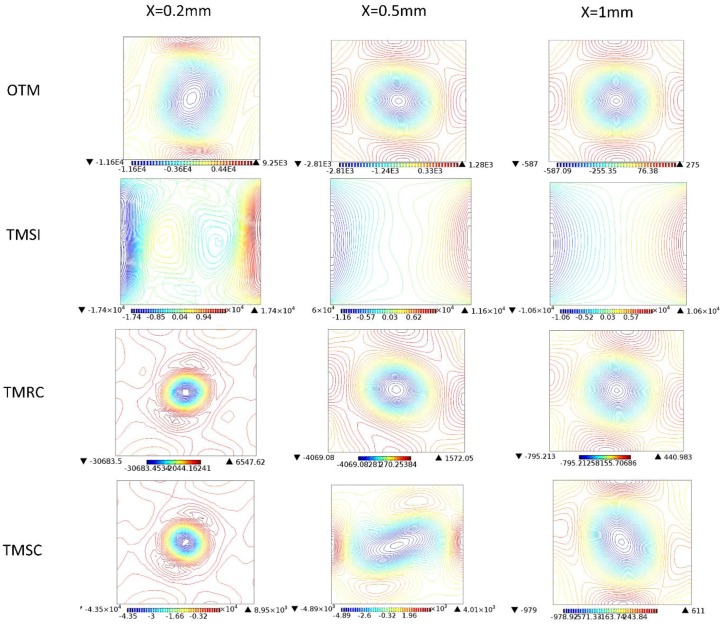
The normal (*x*) vorticity streamline for the four micromixers at *x* = 0.2 mm, 0.5 mm, 1 mm.

Figure 11 show the normal (*x*) vorticity streamline for the four micromixers at *x* = 0.2 mm, 0.5 mm, 1 mm. For OTM, the flow stream gives a better identification of vortex cores, and the normal (*x*) vorticity becomes smaller, changing from *x* = 0.2 mm to 1 mm. However, for TMSI, the flow stream has no vortex core because the obstacles in the two inlets narrow and displace the fluid stream so that they come into the T-junction at different vertical levels, which creates bigger vorticity around the periphery. For TMRC and TMSC, at *x* = 0.2 mm, the flow stream presents bigger and uniform vorticity around the peripheryand very small vorticity in the central. This indicates that, when the fluids flow away from the constriction, it draws away from the channel axis and the swirling flow along the mixing channel decelerates. When reaching a certain position, the fluids achieve rapid mixing. Compared with OTM, the flow streams at *x* = 0.5 mm and 1 mm for TMRC and TMSC have slightly higher vorticity.

### 3.5. Pressure Drop

**Figure 12:**
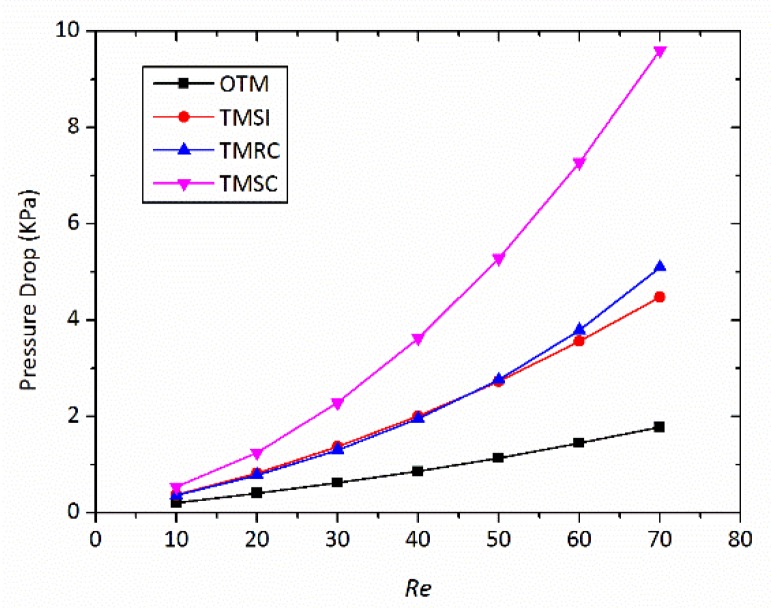
Pressure drop for different micromixer at different Reynolds numbers.

Figure 12 presents the variations in the pressure drop for four micromixers at different Reynolds numbers. Excellent mixing is usually associated with a large pressure drop [45,46]. The large pressure drop causes huge energy loss that will affect the actual results of mixing experiments. Compared to OTM, the pressure drops rapidly with the Reynolds numbers for the three other micromixers. This rapid increase in pressure drop is mainly attributed to the formation of a stronger vortex due to the swirl-inducing inlets and the rectangular constriction. Compared with TMSC with TMRC, the pressure drop caused by the swirl-inducing inlets is slightly higher than by rectangular constriction. TMSC shows the maximum pressure drop throughout the entire Reynolds number range; this indicates that the combination of the swirl-inducing inlets and the rectangular constriction creates a pressure drop. From Figure 12, TMRC is best if situations of the same pressure drop are compared. This is because the pressure drop of TMRC is smaller than that of TMSC, but the mixing index of TMRC is close to that of TMSC.

### 3.6. Micromixer Design

**Figure 13:**
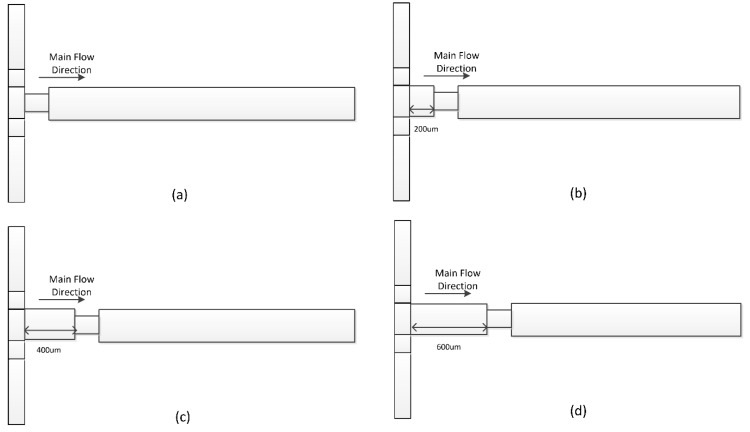
Schematics of (**a**) TMSC, (**b**) TMSC1, (**c**) TMSC2, (**d**) TMSC3.

In this section, the design concept of the TMSC micromixer is introduced. From the preliminary studies on TMSC, the combination of the obstacles in two inlets and the constriction in the mixing channel enhances mixing performance and, at the same time, brings about a large pressure drop. Compared with TMSI, the reason for a larger drop in pressure is that the constriction hinders the flow of the fluid coming from the T-junction. The location of the constriction along the mixing channel is as shown in Figure 13. 

**Figure 14:**
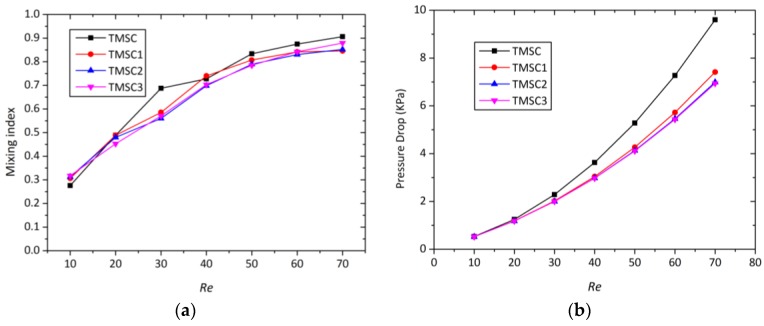
(**a**) Variation of mixing index of micromixer with Reynolds number; (**b**) variation of the pressure drop with Reynolds number.

Figure 14a describes the development of the mixing index with the growth of Reynolds numbers at the surface of *x* = 1 mm. The movement of the constriction along the mixing channel has little adverse effect on its enhancement on mixing index at all Reynolds numbers; for example, at *Re* = 70, the difference between four micromixers in mixing index is no more than 6%. Figure 14b shows the pressure drop variations for four micromixers at various Reynolds numbers. The pressure drop of the three novel micromixers is smaller to TMSC at each Reynolds number. The movement of the constriction along the mixing channel can effectively decrease the pressure drop, and the greater the distance between the constriction and the inlets, the smaller the pressure drop of the micromixer. Micromixer TMSC3 has both the highest mixing index and the smallest pressure drop in three novel micromixers. The reason is that the fluids flow away from the T-junction; the time of free flow is beneficial to the mix and makes the system stable. Of course, the mixing time also decreases with the movement of the constriction along the mixing channel.

## 4. Conclusions

This work proposed three novel 3D micro T-mixers with swirl-inducing inlet and rectangular constriction to enhance the mixing of two fluids. Numerical simulation is used to analyze the mixing performance of three proposed micromixers. The results are compared to the performance of the original 3D micro T-mixer. The flow fields and mixing index are analyzed at Reynolds numbers from 10 to 70. The main findings are as follows.

The swirl-inducing inlet and the rectangular constriction can both enhance mixing. The mixing index of TMSC is the highest among the three micromixers at all the Reynolds numbers tested, especially at *Re* = 70, when the mixing index of TMSC reaches about 91.8%. This indicates that the combination of rectangular constriction and swirl-inducing inlets can produce the high mixing performance at the shortest distance.

For all Reynolds numbers, the mixing index of TMSC along the channel length is the highest among the four micromixers. TMSC can obtain rapid and efficient mixing at a short mixing length.

For TMRC, the rectangular constriction has less impact on the vortex caused by the T-junction channels. However, for TMSC, the rectangular constriction, which impairs the flow from the T-junction to the mixing channel, promotes the development of the vortex. The swirl-inducing inlets give the fluids a better mix, especially at the center of the mixing channel, and the rotational fluids come into the rectangular constriction, which further enhances the mixing effect. For TMSI and OTM, at some distance from the entrance of the mixing channel, the action of convective mixing becomes weak. However, for TMRC and TMSC, after the fluids flow away from the rectangular constriction, the suddenly expanded structure can make the vortex movement active.

This rapid increase in pressure drop is mainly attributed to the formation of a stronger vortex caused by the swirl-inducing inlets and the rectangular constriction. TMSC shows the maximum pressure drop throughout the entire Reynolds number range, from 10 to 70. For the design of TMSC, the movement of the constriction along the mixing channel can effectively decrease the pressure drop.

## Figures and Tables

**Table 1:** Dimensions of different configurations of micromixers.

Type	*W*_3_*/*µm	*H*_3_/µm	*W*_4_/µm	*H*_4_/µm	L_4_/µm
OTM	-	-	-	-	-
TMSI	100	50	-	-	-
TMRC	-	-	100	90	150
TMSC	100	50	90	90	150
